# Nightmares in the general population: identifying potential causal factors

**DOI:** 10.1007/s00127-017-1408-7

**Published:** 2017-07-15

**Authors:** Stephanie Rek, Bryony Sheaves, Daniel Freeman

**Affiliations:** Department of Psychiatry, University of Oxford, Warneford Hospital, Oxford, OX3 7JX UK

**Keywords:** Nightmares, Negative affect, Stressful life events, Cognitive processes

## Abstract

**Background:**

Nightmares are inherently distressing, prevent restorative sleep, and are associated with a number of psychiatric problems, but have rarely been the subject of empirical study. Negative affect, linked to stressful events, is generally considered the key trigger of nightmares; hence nightmares have most often been considered in the context of post-traumatic stress disorder (PTSD). However, many individuals with heightened negative affect do not have nightmares. The objective of this study was to identify mechanistically plausible factors, beyond negative affect, that may explain why individuals experience nightmares.

**Method:**

846 participants from the UK general population completed an online survey about nightmare occurrence and severity (pre-occupation, distress, and impairment), negative affect, worry, depersonalisation, hallucinatory experiences, paranoia, alcohol use, sleep duration, physical activity levels, PTSD symptoms, and stressful life events. Associations of nightmares with the putative predictive factors were tested controlling for levels of negative affect. Analyses were also repeated controlling for levels of PTSD and the recent occurrence of stressful life events.

**Results:**

Nightmare occurrence, adjusting for negative affect, was associated with higher levels of worry, depersonalisation, hallucinatory experiences, paranoia, and sleep duration (odds ratios 1.25–1.45). Nightmare severity, controlling for negative affect, was associated with higher levels of worry, depersonalisation, hallucinatory experiences, and paranoia (*R* ^2^s: 0.33–0.39). Alcohol use and physical activity levels were not associated with nightmares.

**Discussion:**

The study identifies a number of potential predictors of the occurrence and severity of nightmares. Causal roles require testing in future longitudinal, experimental, and treatment studies.

## Introduction

Nightmares are highly dysphoric dreams involving intense negative emotions which primarily present during late-night rapid eye movement (REM) sleep [1]. About one in 20 of the general population experiences nightmares every week [2]. In contrast, estimates of the prevalence of nightmares are far higher for psychiatric populations. For example, frequent nightmares occur in about three-quarters of patients with PTSD [3], about half of patients with borderline personality disorder [4], and at least 10% of patients with schizophrenia [5, 6]. Nightmares are associated with increased psychological distress, self-harm, and suicidal behaviour [4, 7, 8]. A small number of prospective studies indicate that nightmares not only co-occur with psychiatric disorders, but may also be early indicators of the onset of psychotic experiences [9, 10], post-traumatic stress disorder (PTSD) [11] and sleep disturbances [12]. Nightmares often arise following exposure to a traumatic event [13]; they have, therefore, most often been considered in the context of PTSD [14, 15] and are one of the core clinical symptoms of the disorder [16]. Similarly, milder forms of waking life stressors have also been found to precipitate the occurrence of nightmares [17]. For example, research has indicated that a stressful event (e.g. exam stress, natural disasters and death of significant other) can be a cause of more frequent nightmares [17–19]. Negative affect more broadly is also considered a key trigger of nightmares [20], for example, Li et al. [2] found that mood disorders were associated with frequent nightmares (OR 15.57, 95% CI 3.77–64.37) adjusting for a range of potential confounders. Given, however, that many individuals can experience stressors and negative affect without experiencing nightmares, our aim was to identify factors, independent of negative affect (defined as symptoms of anxiety and depression), that may predict the occurrence and severity of nightmares. In this paper, we focus on psychological, psychiatric, and behavioural factors that could plausibly contribute to the triggering of nightmares.

### Cognitive style

Worry is a cognitive style characterised by repetitive negative thinking about future events. It brings fearful and sometimes implausible ideas to mind and keeps them there [21, 22]. Whilst worry is certainly associated with negative affect [23, 24] it may be that nightmares are not accounted for entirely by affect alone. Instead, it is proposed that these repetitive and negatively oriented cognitions (particularly in the pre-sleep period) feed negative dream content, and hence increase the chances of nightmares. This is in line with the continuity hypothesis of dreaming that states dreams reflect waking life experiences, thereby capturing the daytime concerns of the dreamer [25]. It is further proposed that worry not only increases the likelihood of nightmare occurrence but also exacerbates nightmares severity by increasing the daytime preoccupation and distress associated with nightmare experiences, thereby increasing the impairment.

It is, therefore, plausible that a cognitive style such as worry may be a potential causal factor in triggering nightmares and exacerbating nightmare severity, over and above negative affect alone.

### Dissociation

Dissociative experiences such as depersonalisation are relatively common in the general population [26]. One theory is that dissociation arises as a psychological escape (i.e. affect shutdown) triggered by trauma [27]. The shutdown in affect results in a dampening of sympathetic nervous system output and reduced emotional experiencing, mediated by cognitive control mechanisms in frontal brain areas that suppress limbic activity [28, 29]. During REM sleep these frontal areas are relatively de-activated, preventing regulation of the limbic system [30]. This indicates that nightmares may reflect a rebound of suppressed emotional experiences, since psychological escape during sleep is no longer possible.

One influential theory proposes that the dreaming process functions to accomplish fear extinction by exposing the dreamer to the feared stimuli in a safe and an ‘ever-changing sequence of contexts’ [14]. Nightmares, in contrast, would represent a failure of fear memory extinction since the dreaming process is disrupted by awakening from too intense emotional distress. This disruption of the dreaming process will ultimately keep the fear memory alive since fear memory extinction cannot be accomplished and hence increase the likelihood of future nightmare occurrence. It is proposed that the continued avoidance of distressing emotional experiences throughout the day and night will not only increase the likelihood of nightmare occurrence but also increase nightmare severity by increasing the distress. In a study of 116 people in the general population, frequent nightmares were associated with greater levels of dissociation, with nightmare distress having a stronger association with dissociation than nightmare frequency [31].

### Psychotic-like experiences

There are several plausible routes by which nightmares and psychotic experiences may be associated. First, the content of distressing daytime psychotic experiences (i.e. paranoid thoughts and hallucinatory experiences) may be re-experienced during dreams in line with the continuity hypothesis of dreaming [32]. If dreaming about distressing daytime experiences becomes too emotionally intense, the dreamer will likely awaken resulting in a nightmare occurrence. This theory is supported by a recent, case-series of imagery rehearsal therapy (IRT) for nightmares, in which a clinical sample of individuals experiencing psychosis indicated that paranoia and auditory hallucinations reappear during nightmares [33]. Second, dreams and hallucinations in particular are phenomenologically similar: both are characterised by vivid and sensory-rich experiences and a lack of meta-cognitive awareness pointing to similar brain activation patterns during both states [e.g. 34]. Those prone to hallucinations may, therefore, also be particularly prone to frequent dreams and hence at greater risk for nightmares. In addition, low levels of attentional top-down control may result in a rapid, uncritical acceptance of unusual experiences as real and important. It is likely that these same processes are at play in response to nightmare experiences and drive subsequent appraisals. For example, rather than appraising distressing nightmare content as “just a dream”, they may be appraised with a persecutory interpretation such as “the devil sending me a message” or other delusional framework, for example “my dreams predict the future”. Such appraisals likely exacerbate nightmare severity (i.e. preoccupation, distress and impairment in functioning).

Initial research indicates a high prevalence of nightmares in patients with current psychotic experiences [6]. In a student sample, nightmare-related distress and frequency were associated with levels of paranoia and hallucinatory experiences (Spearman’s rho ranged between 0.18 and 0.21) [35] and in a large epidemiological sample, nightmares were longitudinally associated with psychotic experiences (OR 1.62, 95% CI 1.19–2.20) [10]. However, these previous studies have not controlled for other plausible explanatory variables which may account for the association between nightmares and psychotic experiences, specifically negative affect, PTSD symptoms, and stressful life events.

### Behavioural patterns

As nightmares appear during late-night REM sleep, factors influencing this specific sleep stage might increase or decrease the opportunity for nightmares to occur. We highlight three behavioural patterns: alcohol, daytime activity, and sleep duration. Ethanol increases light sleep and amounts of late-night REM sleep [36–38]. Mechanistically, there is evidence that ethanol-induced REM sleep suppression during the first half of the sleep period causes REM sleep rebound in the second half of the night [39] and therefore, longer and more vivid dreams [40] increase the opportunity for nightmares. Greater amounts of daytime activities, particularly physical exercise, might protect against nightmare occurrence by decreasing the amount of total REM sleep [41], whereas poor physical activity might be associated with more frequent nightmares [38]. Preliminary evidence suggests that nightmares occur more frequently with high or low sleep duration [38]. More frequent nightmares were observed among people who slept more than 9 h or less than 7 h per night compared to those who slept 7–9 h. The effect sizes of these associations, however, were small (*χ* ^2^, *p* < 0.001; Cramer *V*, 0.07) and require replication. Mechanistically, a greater length of sleep might increase the amount of late-night REM sleep, thereby increasing the opportunity for nightmares. In contrast, insufficient amount of sleep might be related to physiological hyperarousal, lowering the threshold for awakening.

## The current study

This study aimed to investigate factors associated with nightmare occurrence and severity beyond levels of negative affect. Our primary hypothesis was that factors associated with nightmares would be; worry; depersonalisation; hallucinatory experiences; paranoia; and certain behavioural patterns (alcohol use, longer and shorter sleep duration, activity levels). The strength of each of these associations was assessed whilst controlling for other explanatory variables; negative affect, PTSD symptoms, and recent stressful life events.

## Method

### Participants

The inclusion criteria were; aged between 18 years or older and fluency in the English language. There were no exclusion criteria. We recruited participants from social media advertisements (“Are you interested in your sleep: We are looking for volunteers to complete an online sleep survey”) and from our existing database of individuals who had given consent to be contacted about sleep studies. 1479 individuals responded to the survey advert. To ensure data integrity and the quality of responses, we (1) deleted 602 partial (i.e. incomplete) responses, (2) assessed the time participants needed to complete the survey. Four participants who had response times under 10 min, which was deemed highly unlikely (median completion length 22.6 min), were omitted, and (3) participants’ sleep duration was investigated for unlikely entries (e.g. going to bed after the stated sleep onset time). Thirty one cases were detected and excluded from further analyses. The final sample included 846 participants. Informed consent was obtained prior to participation and the study was approved by the University of Oxford Medical Sciences Research Ethics Committee (R43763/RE001).

### Measures

Nightmare Severity Scale (NSS) [42]. In this scale, a nightmare is defined as a dream with upsetting content which is recalled with distress upon awakening. The NSS assesses nightmare frequency over the past 2 weeks. (“In the past 2 weeks, how many nights (0–14) have you experienced nightmares?”). Moreover, the self-report questionnaire includes 45 items which form a total nightmare severity score. There are three sub-scales which contribute equally to the total score; preoccupation with nightmare experiences [e.g. “It is hard to stop thinking about my nightmare(s)”], nightmare distress [e.g. “I’m distressed by my nightmare(s)”] and impairment in function [e.g. “My nightmare(s) affect my ability to stick to daytime commitments (e.g. work/meeting people/getting to appointments”)]. The severity scale is completed only if the participant reports at least one nightmare. Higher scores indicate greater nightmare severity.

Depression, Anxiety and Stress Scale-21 (DASS-21) [43]. The depression and anxiety scales were used to assess negative affect during the preceding week. The scales have been reported to possess good psychometric properties in clinical and non-clinical samples [44]. Scores on the DASS-21 depression and anxiety sub-scales were summed to provide level of negative affect. Higher scores indicate greater levels of negative affect.

Primary Care-PTSD screen (PC-PTSD) [44]. PTSD symptoms were assessed with the four item PC-PTSD screen. The screen includes an introductory sentence which defines a traumatic event that may have happened (i.e. “In your life, have you ever had any experience that was so frightening, horrible, or upsetting that, in the past month, you:”) without asking participants to identify their own experiences. It contains four questions answered on a binary response scale (yes and no) assessing avoidance and hypervigilance experiences following the experience. The scale has been found to possess good test–retest reliability (*r* = 0.83). The optimally efficient cut-off score for a probable PTSD diagnosis has been found to be 3, with a corresponding sensitivity of 0.78 and a specificity of 0.78 [45]. We used the dimensional score in our statistical analyses.

Social Readjustment Rating Scale **(**SRRS) [46]. This scale assesses stressful life-events over the past year. It entails 43 items and is a self-inventory scale. It lists stressful life events that the participant might have experienced over the past 12 months (e.g. divorce). Each stressful life event on the SRRS has a weighted stress score associated with it (e.g. death of a spouse = 100, whilst minor violation of law = 11) and the total score reflects the sum of these. Scores can, therefore, range from 0 (“no life events experienced”) to 1466 with higher scores indicating greater experience of stressful life events. The temporal stability over a 2-year period has been observed to be high in a non-clinical population [47].

Penn State Worry Questionnaire (PSWQ) [48]. Worry levels were assessed with the 16 items PSWQ which is the most established measure of trait worry style possessing high levels of test–rest reliability in non-clinical samples (*r* = 0.74–0.92) and internal consistency (ranging from 0.88 to 0.95 for clinical and non-clinical samples, respectively; see [49], for a review). Items are rated on a scale ranging from 1 (“Not at all typical for me”) to 5 (“Very typical for me”). Higher scores indicate a greater tendency to worry.

Cambridge Depersonalisation Scale **(**CDS) [50]. This scale is a 29-item self-report questionnaire that assesses the frequency of depersonalisation experiences over the past 6 months. The scale measuring the duration of the experience was omitted. This measurement has been observed to have good psychometric properties [51]. Higher scores indicate greater levels of depersonalisation.

Specific Psychotic Experiences Questionnaire (SPEQ) [51]. Psychotic-like experiences (i.e. hallucinations and paranoia) were measured using subscales from the SPEQ that asking for the frequency of 15 paranoid thoughts and nine hallucinatory experiences. The scales range from 0 (“Not at all”) to 5 (“Daily”). The subscales have been validated as measures of psychotic experiences in a general population sample of adolescents [51]. Higher scores indicate higher levels of paranoia and hallucinatory experiences.

*Alcohol use data* Participants were asked to specify the number of alcoholic drinks they had consumed over the past week. A drink was defined as a (a) regular size serving of beer, (b) regular size glass of wine, or (c) one measure of liquor.

*Munich Chronotype Questionnaire* (MCTQ) [52] We assessed current sleep duration with six selected questions of the MCTO that concern self-reported habitual sleep timings for work and work-free days. A mean sleep duration was calculated for each participant by weighting sleep duration by the amount of work and free days, respectively.

*Borg’s CR10 Scale* [53] This scale was used as a psychophysical tool to assess subjective perception of effort during physical activities over the past week. Participants were asked to indicate the intensity of exercise (1 = no activity (e.g. watching TV, reading a book, driving a car, etc.) to 10 = very very hard (e.g. running a race) they had completed each day over the past 7 days. An increase in power, heart rate and oxygen uptake has been shown to correspond to an increase in subjective estimations in relation to physical effort using the Borg’s CR10 Scale [54]. Participants rated the maximum intensity activity they had completed each day. The total score across the 7 days was used as an indicator for physical activity levels.

*Insomnia Severity Index* **(**ISI) [55] The ISI is a seven item self-report instrument measuring insomnia severity. The items relate to the previous month. The questionnaire has been found to be a reliable and valid measure to quantify perceived insomnia severity [55]. A cut-off score of 10 has been suggested for detecting insomnia cases with of sensitivity 86.1% and a specificity of 87.7% in the community sample [56]. Higher scores indicate greater insomnia symptom severity.

#### Demographics

Participants were asked to indicate their age, gender, nationality, work status, marital status, alcohol consumption, cannabis or cocaine use.

## Procedure

Participants were invited to take part in a cross-sectional online survey, which was programmed using Qualtrics software. Informed consent was obtained online at the start. In the first step of the survey, participants provided demographic information. Subsequently, questionnaires were presented in the following order: DASS-21, PSWQ, ISI, SPEQ, CDS, NSS, PC-PTSD, MCTQ, Borg’s CR10 Scale and SRRS. Finally, participants were asked to enter their email address to be included in a prize draw.

### Statistical analysis

All analyses were carried out using the statistical package for social sciences version 20 [57].

The survey questions were mandatory and the very small amount of missing data resulted from rare failings of the software. There were missing items for 11 individuals for the activity measure, which were replaced with the participant’s mean activity level from the other days over the past week. Further, missing values on alcohol (*n* = 20), cannabis (*n* = 3) and cocaine use (*n* = 3) were handled using median imputations. Results did not change as a consequence of these imputation procedures.

First, descriptive statistics and the strength of statistical association between variables were tested by bivariate Pearson’s correlation coefficients, Chi-square tests, and independent samples *t* tests when appropriate. Here, participants who experienced at least one nightmare over the past fortnight were compared to controls (i.e. the reference group not experiencing nightmares). We report magnitudes of effect sizes according to Cohen [58]: correlation coefficients of 0.10 are considered “small”, those of 0.30 are “medium”, and those of 0.50 are “large”.

To test the hypotheses concerning nightmare occurrence, nightmare frequency over the past fortnight was dichotomised (no nightmares = 0, one or more nightmares = 1) and used as the dependent variable. We ran multiple binary regression analyses on nightmare occurrence and multiple linear regression analyses on log-transformed nightmare severity. In both analyses, negative affect was added as an independent variable and one of the following variables was added separately to the model in the first stage; worry, depersonalisation, hallucinatory experiences, paranoia, alcohol use, sleep duration, and activity levels. To assess the predictive ability of shorter and longer sleep duration, we tested the quadratic function for significance. In the second step PTSD symptoms and thereafter stressful life events were added as additional independent variables to the models. All independent variables were standardised to ease the interpretation of odds ratios (ORs) and standard regression coefficients. For the interpretation of results it should be remembered that for continuous scales with standardised values the ORs refer to the risk-increase for a 1-standard deviation increase in the independent variable. All hypothesis testing was two-tailed. 95% confidence intervals (CI), ORs, and *R* ^2^ are reported when appropriate.

In our primary analysis, we deliberately included no covariates other than negative affect in our data analysis on nightmare occurrence and severity. Miller and Chapman [59] caution on including covariates in statistical analyses, particularly in studies with a non-randomised study design. Here, overlap or truly shared variance between variables can only be artificially controlled for and may result in inappropriate removal of variance. The study was designed to identify variables associated with nightmare occurrence and severity beyond negative affect only. However, PTSD symptoms and life events were included as additional independent variables in our secondary tests as there were strong grounds for their use.

## Results

### Demographics

**Fig. 1:**
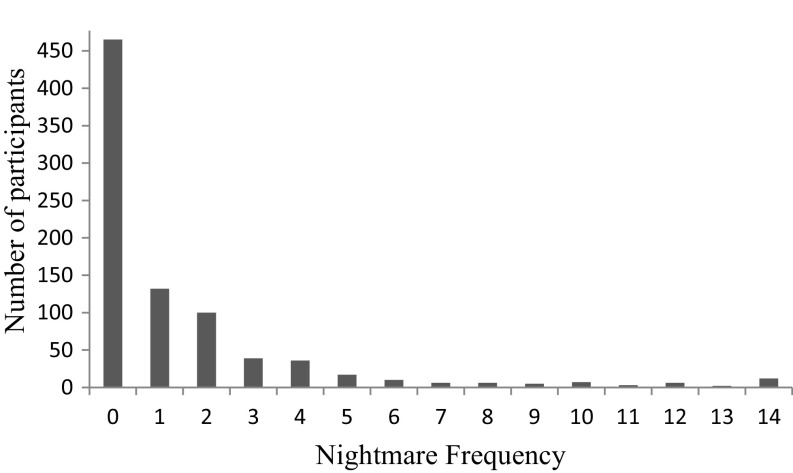
Nightmare frequency in the sample over the past two weeks

Eighty nine percent of the sample was female and the mean age was 44 years (SD 15.9), range 18–77 (see Table 1). 381 individuals (45%) reported experiencing at least one nightmare over the past fortnight. The distribution of nightmare occurrence was highly skewed with about half of the participants indicating no nightmare experiences and fewer participants indicating more than one nightmare over the past fortnight (see Fig. 1). The mean nightmare severity score was 80.01 (SD 31.12), range 45–221.

**Table 1:** Demographics of participants with nightmares compared to the control group (i.e. those without nightmares)

	Nightmares (*n* = 381)	Control group (*n* = 465)	*χ* ^2^	*df*	*p*
Sex, *n*					
Male	39	55	0.5	1	0.464
Female	342	410			
Ethnicity, *n*					
White	358	450	3.8	1	0.050
Any other	23	15			
Cocaine, *n*					
Yes	22	15	3.24	1	0.071
No	359	450			
Cannabis, *n*					
Yes	70	47	12.00	1	0.001**
No	311	418			
Insomnia, *n*					
Not clinically significant	66	185	50.64	1	<0.001***
Clinically significant	315	280			

	Nightmares (*n* = 381)	Control group (*n* = 465)	Odds ratio	*p*	95% CI
Marital status					
Single	129	125	Reference		
Cohabiting	60	46	1.264	0.314	0.801, 1.994
Married	155	221	0.680	0.018*	0.493, 0.936
Divorced	32	55	0.564	0.025*	0.342, 0.930
Widowed	5	18	0.269	0.012*	0.097, 0.747
Employment status, *n*					
Employed	231	294	Reference		
Retired	43	75	0.730	0.134	0.483, 1.102
Student	80	67	1.520	0.026*	1.052, 2.195
Housewife/husband	16	13	1.566	0.242	0.739, 3.322
Unemployed	11	16	0.875	0.739	0.398, 1.922

* *p* < 0.05, ** *p* < 0.01, *** *p* < 0.001

### Unadjusted associates of nightmares

Individuals with nightmares reported higher levels of insomnia and a greater likelihood of having used cannabis compared to those without nightmare experiences. Individuals who were married, divorced, or widowed were less likely to experience nightmares compared to those who were single. Students had an increased likelihood of experiencing nightmares compared to full-time employed participants (Table 1).

Independent samples t tests found that the nightmare group was significantly younger and reported significantly higher levels of negative affect, worry, depersonalisation, hallucinatory experiences, paranoia, compared to the individuals not experiencing nightmares. Cohen’s *d* effect sizes ranged from low to high (Table 2). Nightmare occurrences was unrelated to sex and ethnicity.

**Table 2:** Independent samples* t* tests between nightmare occurrence and control group (i.e. not experiencing nightmare) for all variables

Variable	Nightmares (*n* = 381) Mean (SD)	Control group (*n* = 465) Mean (SD)	*t*	ES (*d*)	*df*	*p*
Age	41.4 (16.1)	46.8 (15.3)	5.4	0.3	844	<0.001***
Negative affect	48.1 (16.5)	39.4 (11.9)	−8.9	0.6	844	<0.001***
Worry	52.8 (13.9)	44.4 (14.1)	−8.3	0.6	844	<0.001***
Depersonalisation	46.4 (16.6)	38.4 (12.4)	−8.0	0.5	844	<0.001***
Hallucination	15.3 (8.1)	12.1 (5.2)	−3.6	0.5	844	<0.001***
Paranoia	31.2 (17.6)	23.7 (12.3)	−7.5	0.5	844	<0.001***
Alcohol use	20.0 (9.2)	20.1 (8.5)	0.2	<0.1	844	0.832
Activity level	15.5 (4.3)	15.6 (4.1)	0.1	<0.1	844	0.806
Sleep duration	7.6 (1.3)	7.3 (1.2)	−0.3	0.2	844	0.002**
PTSD	1.3 (1.5)	0.7 (1.1)	−0.6	0.7	844	<0.001***
Life events	223.6 (151.5)	163.1 (122.3)	−60.5	0.4	844	<0.001***

* *p* < 0.05, ** *p* < 0.01, *** *p* < 0.001

Bivariate Pearson’s correlations between nightmare severity and the independent variables were highly significant. Here, higher levels of nightmare severity were associated with higher ratings of negative affect, worry, depersonalisation, hallucination, paranoia, sleep duration, PTSD symptoms, and life events. Alcohol use and activity levels were unrelated to nightmare severity (Table 3).

**Table 3:** Correlations between nightmare severity and the independent variables

Variable	Nightmare severity
Negative affect	0.56, *p* < 0.001***
Worry	0.45, *p* < 0.001***
Depersonalisation	0.58, *p* < 0.001***
Hallucination	0.49, *p* < 0.001***
Paranoia	0.52, *p* < 0.001***
Alcohol use	0.01, *p* = 0.810
Sleep duration	−0.14, *p* = 0.005**
Activity level	−0.06, *p* = 0.280
PTSD	0.50, *p* < 0.001***
Life events	0.26, *p* < 0.001***

* *p* < 0.05, ** *p* < 0.01, *** *p* < 0.001

### Adjusted analysis controlling for negative affect, PTSD symptoms and stressful life events

#### Worry

Worry was significantly associated with nightmare occurrence when negative affect was controlled (see Table 4). This remained significant after including PTSD symptoms and stressful life events. Nightmare severity was also significantly associated with worry, controlling for negative affect, and remained significant after including PTSD symptoms and thereafter stressful life events in the analysis (Table 5).

**Table 4:** Adjusted associations of nightmare occurrence with worry, depersonalisation, hallucination, paranoia, alcohol use, sleep duration, and activity levels

	Adjusted for negative affect			Adjusted for negative affect and PTSD symptoms			Adjusted for negative affect, PTSD symptoms, and life events		
	Odds ratio	*p*	95% CI	Odds ratio	*p*	95% CI	Odds ratio	*p*	95% CI
Worry	1.45	<0.001***	1.21, 1.73	1.40	<0.001***	1.16, 1.17	1.38	0.001**	1.15, 1.66
Depersonalisation	1.33	0.006**	1.08, 1.63	1.25	0.037*	1.01, 1.54	1.21	0.073	0.98, 1.50
Hallucination	1.40	<0.001***	1.17, 1.67	1.34	0.001**	1.12, 1.61	1.30	0.004**	1.09, 1.56
Paranoia	1.25	0.020*	1.04, 1.51	1.18	0.100	0.97, 1.43	1.14	0.185	0.94, 1.39
Alcohol use	0.99	0.370	0.98, 1.01	0.99	0.450	0.98, 1.01	0.99	0.510	0.98, 1.01
Sleep duration	1.34	<0.001***	1.15, 1.55	1.35	<0.001***	1.16, 1.57	1.35	<0.001***	1.16, 1.58
Activity level	1.05	0.550	0.91, 1.21	1.04	0.600	0.90, 1.20	1.02	0.730	0.89, 1.19

* *p* < 0.05, ** *p* < 0.01, *** *p* < 0.001

**Table 5:** Adjusted associations of nightmare severity with worry, depersonalisation, hallucination, and paranoia

	Adjusted for negative affect					Adjusted for negative affect and PTSD symptoms					Adjusted for negative affect, PTSD symptoms, and life events				
	*b*	SE	*p*	*R* ^2^	95% CI	*b*	SE	*p*	*R* ^2^	95% CI	*b*	SE	*p*	*R* ^2^	95% CI
Worry	0.07	0.018	<0.001***	0.33	0.03, 0.10	0.06	0.018	0.004**	0.41	0.02, 0.09	0.06	0.017	0.004**	0.41	0.02, 0.09
Depersonalisation	0.12	0.016	<0.001***	0.39	0.08, 0.15	0.10	0.016	<0.001***	0.45	0.06, 0.13	0.09	0.016	<0.001***	0.45	0.06, 0.13
Hallucination	0.09	0.013	<0.001***	0.39	0.06, 0.11	0.08	0.012	0.001**	0.45	0.05, 0.10	0.08	0.012	0.001**	0.45	0.05, 0.10
Paranoia	0.09	0.015	<0.001***	0.36	0.06, 0.11	0.06	0.015	<0.001***	0.42	0.03, 0.09	0.06	0.015	<0.001***	0.42	0.03, 0.09

* *p* < 0.05, ** *p* < 0.01, *** *p* < 0.001

#### Depersonalisation

Depersonalisation was significantly associated with nightmare occurrence controlling for negative affect. The significance level was slightly reduced but still significant after including PTSD symptoms in the analysis. When stressful life events were also included depersonalisation was no longer significantly associated with nightmare occurrence (Table 4). Depersonalisation was significantly associated with nightmare severity controlling for negative affect, PTSD symptoms, and stressful life events (Table 5).

#### Psychotic-like experiences

Nightmare occurrence was significantly associated with both hallucinations and paranoia over and above negative affect. After including PTSD symptoms and stressful life events, hallucinations remained highly significant; however, paranoia was not significantly associated with nightmare occurrence anymore (Table 4). Both hallucinations and paranoia were significantly associated with nightmare severity controlling for negative affect, PTSD symptoms, and stressful life events (Table 5).

#### Alcohol use, physical activity, and sleep duration

Longer, but not shorter, sleep duration was significantly associated with nightmare occurrence controlling for negative affect. This effect still remained after inclusion of PTSD symptoms and stressful life events in the model (Table 4). Alcohol use and activity levels were not associated with nightmare occurrence.

The same pattern of results for all analyses was obtained when additionally adjusting for age and the nightmare severity variable was not log transformed.

## Discussion

This is the first study to investigate a wide range of psychological, psychiatric, and behavioural factors that could plausibly contribute to nightmare occurrence and nightmare severity over and above more established causal factors (negative affect, PTSD symptoms, and stressful life events). Higher levels of worry, hallucinatory experiences and longer sleep duration were significantly associated with whether or not participants experienced nightmares, even after controlling for negative affect, PTSD, and stressful life events. Worry, depersonalisation, paranoia and hallucinatory experiences were significantly associated with the severity of nightmares. Again, these associations remained significant after controlling for negative affect, PTSD symptoms, and stressful life events. The key caveat is the cross-sectional design, preventing causal interpretations.

Of the variables tested, worry was the strongest associate of the occurrence of nightmares, and also related to the severity rating of nightmares. Given that these associations held after controlling for negative affect, it seems plausible that the amplification of negative thought content, generated by this cognitive style is associated both with the occurrence of nightmares and also the resultant distress, preoccupation and impairment that they cause. An important next step is to assess the direction of the relationship between worry and nightmares. It is most likely that pre-sleep worry increases the likelihood of distressing nightmares, and, in turn, nightmares may trigger worry the following day, resulting in a self-perpetuating circle. It is, therefore, possible that the relation between nightmares and worry is bidirectional. Given that effective interventions already exist for the treatment of worry [60, 61], one approach towards assessing causality would be to treat worry and assess the impact on nightmare occurrence and severity (termed a causal interventionist approach, e.g. [62]). If successful, worry may be a novel treatment target to boost the efficacy of nightmare interventions.

Longer sleep duration showed a strong and consistent association with nightmare occurrence, even after adjustments for negative affect, PTSD symptoms, and stressful life events. Here, a longer sleep duration might increase the amount of late-night REM sleep [38, 63], thereby increasing the opportunity for nightmares to occur. We predict that stabilisation of the sleep window (i.e. reducing a long sleep duration) might thus lead to a reduction in nightmare occurrence for those with longer sleep durations.

More broadly, our findings indicate that nightmares may not be wholly accountable to negative affect, PTSD symptoms, and stressful life events. Hallucinatory experiences predicted nightmare occurrence and both depersonalisation and psychotic-like experiences also predicted nightmare severity. Clearly, further research is needed, ideally in experimental or treatment study designs, to assess the direction of causality between associations and test possible mechanistic links. Nonetheless, this study has identified novel processes that may be predictive of nightmare occurrences and severity.

Surprisingly, no associations were found between nightmare occurrences and alcohol use or activity levels. Limitations in our measures may be a potential reason for earlier results not being replicated (e.g. [38]). We did not assess the duration and time of the day of alcohol consumption but only the dose over the past week. Similarly, with regards to physical activity, we did not differentiate between acute and regular exercise, assessed baseline physical activity levels, the duration of exercise bouts, nor the time of the day when performed. All of these variables, however, might affect REM sleep [64] and thereby, the likelihood to experience nightmares. More in-depth assessments of alcohol use and physical activities on nightmare experiences are warranted to arrive at firm conclusions.

## Limitations and directions for future research

There are clear limitations of the study. Chiefly, the cross-sectional design limits the strength of any causal interpretations. Moreover, the sample is not representative of the general population. Ninety percent of the sample was female and the prevalence of nightmares was higher than in epidemiological studies. Since the study was advertised as a “sleep survey about dreams and nightmares”, it is probable that participants with sleep problems were more likely to complete our study [supported by the high level of clinically significant insomnia in our sample (70%)]. In addition, the high nightmare prevalence may be due to the duration nightmares were assessed for (i.e. 2 weeks instead of weekly prevalence). While these differences limit the generalisability of our findings, the current study benefitted to some degree from the non-representativeness. The study was not set up to assess the prevalence rate of nightmares but rather to identify risk factors beyond negative affect that may predict nightmares. Therefore, the high number of individuals reporting nightmare problems gave sufficient power to test the associations. However, future studies will benefit from more representative samples (e.g. using patient populations).

Notwithstanding these caveats, the current study shows novel cross-sectional associations between psychological, psychiatric, and behavioural factors with nightmares, which were not accounted for by negative affect, PTSD symptoms, and stressful life events alone. Instead, for example, levels of worry and sleep duration showed strong and consistent associations with the occurrence of nightmares. Clearly, causal relationships need testing in future research using more representative samples.
